# Emergency Nurses' Compliance with Standard Precautions during the COVID-19 Pandemic at Governmental Hospitals in Hail City, Kingdom of Saudi Arabia

**DOI:** 10.4314/ejhs.v33i1.4

**Published:** 2023-01

**Authors:** Bahia Galal A Hassan Siam, Ohoud Awadh Suwaimil ALreshidi

**Affiliations:** 1 Assistant Professor of Medical-Surgical Nursing, College of Nursing, Hail University, Saudi Arabia; 2 Master Degree in Emergency Nursing, College of Nursing, Hail University, Saudi Arabia

**Keywords:** Compliance, COVID-19 pandemic, Emergency departments, Nurses, Saudi Arabia, Standard precautions

## Abstract

**Background:**

This study was conducted to assess nurses' compliance with standard precautions during COVID-19 pandemic at emergency departments, Hail city, Saudi Arabia.

**Methods:**

A cross-sectional study was conducted in the year 2021, at emergency departments of governmental hospitals in Hail city, Saudi Arabia. A total of 138 emergency nurses were selected using a census sampling method, and included in the current study. Of them, 56(40.6%) were from King Khalid Hospital, 35(25.4%) from King Salman Specialist Hospital, 28(20.3%) from Sharaf Urgent Care Hospital, and 19(13.8%) from Maternity and Child Hospital. The compliance with standard precautions scale was used, and socio-demographic characteristics were assessed using a structured questionnaire. Statistical analysis was performed using SPSS version 28.

**Results:**

A large percentage (71.0%) of the studied nurses were females, and (78.3%) were Saudi. The mean scores of compliances with standard precautions ranged from 3.1 to 3.9 out of 4. The overall compliance rate with all components of standards precautions was optimal (92.75%). Significant statistical differences were found in the mean scores of the “prevention of cross infection from person to person” with age; and between the mean scores of the “decontamination of spills and used article” with profession carrier P-values = 0.013, and 0.016, respectively.

**Conclusions:**

The compliance with standard precautions by emergency nurses was optimal (more than 90%). The mean compliance scores with the standard precautions could be associated with age and professional category. Continuous training program to enhance compliance with standard precautions among emergency nurses with continuous follow up and evaluation are recommended.

## Introduction

On 1^st^ February 2020, Coronavirus disease (COVID-19) was designated as a public health emergency of international concern by the World Health Organization (WHO). It has become a global pandemic, and, on 30 April 2022, there have been 511,965,711 confirmed cases of COVID-19 worldwide, including 6,240,619 deaths (1). In Saudi Arabia, the first positive case was reported by the Ministry of Health (MOH) on 2^nd^ March 2020, and the number doubled in the country within one month, representing a critical challenge for healthcare professionals (2, 3). The widespread and rapid transmission of the COVID-19 has led the WHO to declare an international public health emergency state (1). The COVID-19 is transmitted through direct contact with the infected person's respiratory droplets and contact with infected surfaces and can survive for hours on the surface (4, 5). The Kingdom of Saudi Arabia began taking an early precautionary action for COVID-19 pandemic. This was due to the belief that starting earlier would prevent a sharp increase in the number of cases in Saudi Arabia and prevent COVID-19 from becoming an epidemic within the country (6). In healthcare settings, nurses are at risk of acquiring infection through occupational exposure in different healthcare settings than general population in the community. During the COVID-19 outbreak, many healthcare workers have been infected with COVID-19 worldwide (7,8).

Standard precautions are a set of infection control practices that healthcare personnel use to reduce transmission of microorganisms in healthcare settings; it is a very important principle and practice among health workers globally (9, 10). Standard precautions, published by the Center for Disease Control and Prevention (CDC), apply to all environments and cases where healthcare workers contact patients. It is a key component of healthcare-associated infection control for the primary prevention of the spread of blood borne and other pathogens. Other components include hand hygiene, appropriate use of personal protective equipment, safe use and disposal of injection needles, elimination of environmental and equipment contamination, patient placement, and management of textiles and wastes (11). By complying with standard precautions, nurses can prevent exposure to potential infection sources, thus ensuring both their patients' safety and their own (12).

In addition, compliance to standard precautions in all situations would be one of the most effective methods to minimize the cross-transmission, regardless of suspected or confirmed infection status of the patients especially under pandemics. As a result, all feasible precautions must be done to prevent the transmission of infection (13, 14). Nurses have critical roles and responsibilities during the COVID-19 pandemic. They will continue to be at the front lines of patients care in hospitals and actively involved with evaluation and monitoring in the community (15). They are, however, at a higher risk of being infected, which might make epidemic control difficult and result in the healthcare system collapsing (16). However, despite standard precautions being carried out for more than a decade in healthcare settings, which has resulted in a considerable decline of healthcare associated infections, low compliance rates with standard precautions among healthcare professionals remains a challenge (10,12). For example, a study conducted among nursing students in Saudi Arabia reported an observed adherence of 60.1% (17). Therefore, it is crucial to investigate the emergency nurses' compliance with standard precautions during the COVID-19 pandemic. The aim of the current study was to assess the rate of emergency nurses' compliance with standard precautions during the COVID-19 pandemic at governmental hospitals in Hail city, Kingdom of Saudi Arabia.

## Methods

**Study design and setting**: A cross-sectional study was conducted, at emergency departments of the four governmental hospitals (King Khalid, King Salman Specialist, Sharaf Urgent Care, and Maternity and Child Hospitals) in Hail city of Saudi Arabia.

**Study population**: The current study included all nurses, both genders, aged 20 to 60 years, and any nationality who worked at the four governmental hospitals in Hail city of Saudi Arabia.

**Sample size determination**: The total number of nurses working at the emergency departments of the four governmental hospitals (King Khalid, King Salman Specialist, Sharaf Urgent Care, and Maternity and Child Hospitals) in Hail city of Saudi Arabia was 138 nurses. All emergency nurses (138 nurses), both genders, aged 20 to 60 years, selected by a census sampling method were included in the current study.

**Study period**: The study was conducted within three months (from the beginning of October 2021 to the end of December 2021).

**Data collection**: A structured questionnaire consisted of two parts was used: Part I: The Nurses' socio-demographic data sheet including age, nationality, gender, educational level, profession career and years of experience; and Part II: The Compliance with Standard Precautions Scale (CSPS): A structured questionnaire developed by Lam et al. (2011), it consists of 20 items covering five dimensions of standard precautions, 16 positively worded items, and 4 negatively worded items (item 2, 4, 6, and 15) (18). In addition, permission was obtained from Dr. Simon Ching LAM to use the tool. Furthermore, the CSPS-Arabic version was used as a reference during translation of the original CSPS tool. The CSPS-Arabic version is a valid and reliable tool for measuring the compliance with standard precautions, and can be employed by researchers to conduct meaningful studies evaluating compliance with standard precautions in Saudi Arabia and in other Arabic speaking countries (19). The dimensions of CSPS are grouped based on the standard precautions' guidelines and in its items that address issues related to clinical practice. The dimensions are grouped as follows: Prevention of cross infection from person to person (PCIP2P): 7 items (items 1, 2, 3, 8, 9, 11, and 12); use of protective device (UPD): 6 items (items 7, 10, 13, 14, 15, and 16); disposal of sharps (DS): 3 items (items 4, 5, and 6); decontamination of spills and used article (DSUA): 3 items (items 18, 19, and 20); and disposal of waste (DW): 1 item (item 17) (18).

**Scoring system**: A four-point Likert scale ranged from (one “never” to four “always”). Items 2, 4, 6, and 15 are negatively stated. Thus, scores reversed before computations. The total score ranged from “1” to “80”. The mean score for each item is considered out of “4”. The total compliance rate refers to the average compliance with all 20 items in percentage. It is optimal when compliance rate is >90%, satisfactory between 80%-89%, suboptimal (unsatisfactory) between 50%-79%, and poor compliance for less than 49% (18). The item compliance rate refers to the mean score of each item, rate estimated by average mean and total per each item.

**Test of reliability**: In the present study, the reliability of CSPS was measured using Cronbach's Alpha, and the value was “0.824” which indicates high reliability. Additionally, pilot study was carried out on twenty participants to enable the researcher to examine the tools of the study.

**Statistical analysis**: The Statistical Package for the Social Sciences (SPSS) version 28.0 software was used for all statistical analyses. All categorical variables were presented as frequencies and percentages, while continuous variables were presented as means and standards of deviation. The used tests were independent t-test and one-way analysis of variance (ANOVA). P-values that are <0.05 was considered significant.

**Ethical approval**: The study protocol was approved by the Scientific Research Ethical Committee of Hail University, the hospitals directors, and the Ethics Committee for Bioethics Research in Hail Health (No. 2021/45), as well as approval letter was obtained from Hail hospitals. Additionally, a questionnaire with a statement of consent on the front page was obtained from each participant without force prior to the study after reading a statement that provided a full explanation of the study's purpose. In addition, the protection of the privacy and confidentiality of the research participants was ensured. Moreover, the researchers coordinated with the nursing offices at the emergency departments of the four included governmental hospitals and acquired the schedules of the emergency nurses. Then, researchers visited the emergency nurses during their break time to invite them to participate in the study, and the data collection process was conducted without any restrictions.

## Results

A total of 138 emergency nurses were selected using a census sampling method, and included in the current study. Of them, 56 (40.6%) nurses were from King Khalid Hospital, 35 (25.4%) nurses from King Salman Specialist Hospital, 28 (20.3%) nurses from Sharaf Urgent Care Hospital, and 19 (13.8%) nurses from Maternity and Child Hospital (Table 1). Additionally, Table 1 reveals that 40.6 % of the studied nurses were working in King Khalid Hospital. 51.4% of the study participants were in age group (35 to 45 years). 71.0 % were females, and 78.3% were Saudi. Regarding educational level and profession carrier, 80.4%, 79.7, of the nurses had bachelor in nursing, and were staff nurses, respectively. 49.3 % of the studied nurses had 5 to 10 years working experience.

**Table 1:** Socio-demographic data of the emergency nurses

Variables	Frequency (n=138)	Percentage (%)
**Hospital name**		
King Khalid Hospital	56	40.6
King Salman Specialist Hospital	35	25.4
Sharaf Urgent Care Hospital	28	20.3
Maternity and Child Hospital	19	13.8
**Age (years)**		
20 to < 35 years	43	31.2
35 to 45 years	71	51.4
More than 45 years	24	17.4
**Gender**		
Male	40	29.0
Female	98	71.0
**Nationality**		
Saudi	108	78.3
Non-Saudi	30	21.7
**Educational level**		
Diploma in nursing	21	15.2
Bachelor in nursing	111	80.4
Master degree in nursing	6	4.3
**Profession career**		
Nursing staff	110	79.7
Profession career	28	20.3
**Years of experience**		
Less than 5 years	41	29.7
From 5 to 10 years	68	49.3
More than 10 years	29	21.0

Data are expressed as percentage for categorical variables.

In addition, Table 2 shows the mean scores and rate of compliance of the emergency nurses with the standards precautions. The results revealed that the mean scores of compliances with the standard precautions of the studied nurses ranged from 3.1 to 3.9 out of 4. The total compliance rates of prevention of cross infection from person to person, use of protective device, disposal of sharps, decontamination of spills and used article, and disposal of waste were 92.8, 94.2, 87.5, 96.5, and 87.5 respectively, with overall compliance rate with all components of standards precautions scores was 92.75. Furthermore, for sub-domains, the results revealed that the compliance rate is slightly lower as regards to “using only water for hand washing” however it is satisfactory (85.0%); less compliant with reusing a surgical mask or disposable personal protective equipment (87.5%); and less compliant with “the sharps box is only disposed when it is full” (77.5%) as shown in Table 2.

**Table 2:** Mean scores and rate of compliance of the emergency nurses with the standards precautions

Items		Frequency Standards Precautions Scores (n=138)					Compliance Rate (%)

		Always	Sometimes	Seldom	Never	Mean scores	
**Prevention of Cross Infection from Person to Person (PCIP2P)**							
1	I wash my hands between patient contacts.	120(87)	12 (8.7)	5 (3.6)	1(0.7)	3.8	95.0
2	I only use water for hand washing.	19(13.8)	13 (9.4)	5 (3.6)	101(73.2)	3.4	85.0
3	I use alcohol hand rubs as an alternative if my hands are not visibly soiled.	103(74.6)	27 (19.6)	3 (2.2)	5 (3.6)	3.7	92.5
4	I take a shower in case of extensive splashing even after I have put on Personal Protective Equipment.	91 (65.9)	39 (28.3)	5 (3.6)	3 (2.2)	3.6	90.0
5	I cover my wound (s) or lesion (s) with waterproof dressing before patient contacts.	121(87.7)	10 (7.2)	7 (5.1)	0 (0.0)	3.8	95.0
6	I change gloves between patient contacts.	129(93.5)	5 (3.6)	2 (1.4)	2 (1.4)	3.9	97.5
7	Decontaminate my hands immediately after removal of gloves.	117(84.8)	18 (13.0)	2 (1.4)	1 (0.7)	3.8	95.0
**Total PCIP2P:**						**3.7**	**92.8**
**Use of Protective Device (UPD)**							
1	I remove Personal Protective Equipment in a designated area.	115(83.3)	16 (11.6)	4 (2.9)	3 (2.2)	3.8	95.0
2	I wear gloves when I am exposed to body fluids, blood products, and any excretion of patients.	123(89.1)	12 (8.7)	1 (0.7)	2 (1.4)	3.9	97.5
3	I wear a surgical mask alone or in combination with goggles, face shield and apron whenever there is a possibility of a splash or splatter.	120(87.0)	12 (8.7)	4 (2.9)	2 (1.4)	3.8	95.0
4	My mouth and nose are covered when I wear a mask.	120(87.0)	14 (10.1)	2 (1.4)	2 (1.4)	3.8	95.0
5	I reuse a surgical mask or disposable Personal Protective Equipment.	17 (12.3)	7 (5.1)	5 (3.6)	109(79.0)	3.5	87.5
6	I wear a gown or apron when exposed to blood, body fluids or any patient excretions.	120(87.0)	11 (8.0)	4 (2.9)	3 (2.2)	3.8	95.0
**Total UPD:**						**3.8**	**94.2**
**Disposal of Sharps (DS)**							
1	I recap used needles after giving an injection.	19 (13.8)	9 (6.5)	3 (2.2)	107(77.5)	3.4	87.5
2	I put used sharp articles into sharps boxes.	128(92.8)	9 (6.5)	0 (0.0)	1 (0.7)	3.9	97.5
3	The sharps box is only disposed when it is full.	19 (13.8)	27 (19.6)	7 (5.1)	85 (61.6)	3.1	77.5
**Total DS:**						**3.5**	**87.5**
**Decontamination of Spills and Used Article (DSUA)**							
1	I decontaminate surfaces and equipment after use.	125(90.6)	10 (7.2)	2 (1.4)	1 (0.7)	3.9	97.5
2	I wear gloves to decontaminate used equipment with visible soils.	124(89.9)	10 (7.2)	4 (2.9)	0 (0.0)	3.9	97.5
3	I clean up spillage of blood or other body fluid immediately with disinfectants.	119(86.2)	16 (11.6)	3 (2.2)	0 (0.0)	3.8	95.0
**Total DSUA**						**3.9**	**96.5**
**Disposal of Waste (DW)**							
1	Waste contaminated with blood, body fluids, secretion, and excretion are placed in red plastic bags irrespective of patient's infective status.	105 (76.1)	9 (6.5)	7 (5.1)	17 (12.3)	3.5	87.5
**The overall compliance**						**3.7**	**92.75**

Data are expressed as percentage for categorical variables. The item compliance rate refers to the mean score of each item, rate estimated by average mean and total per each item.

**Figure 1:**
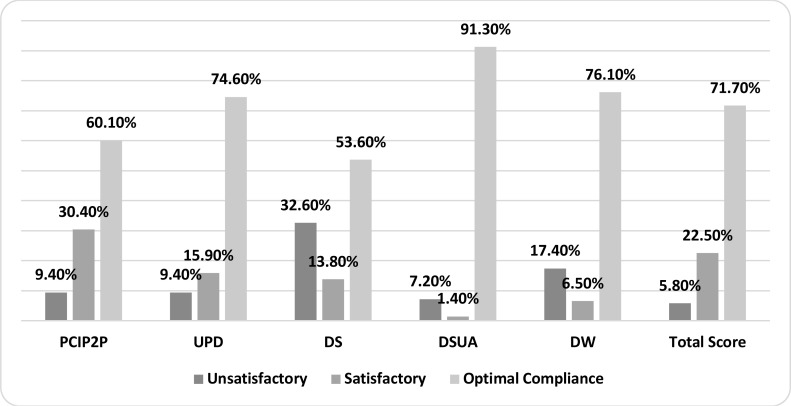
Nurses' compliance scores with the standard precautions PCIP2P: Prevention of Cross Infection from Person to Person; UPD: Use of Protective Device; DS: Disposal of Sharps; DSUA: Decontamination of Spills and Used Article; DW: Disposal of Waste

Moreover, Figure 1 shows that optimal satisfaction represents the highest compliance rate of the total score and all sub-dimensions of the standard precaution scale. Additionally, Table 3 reveals that there is a significant statistical difference in the mean scores of the “prevention of cross infection from person to person” with age (P-value=0.013), and, the highest mean was for the age group from 35 to 45 years (middle age). In addition, a significant statistical difference was found in the mean scores of the “decontamination of spills and used article” with profession carrier (p values=0.016).

**Table 3:** Relation between socio-demographic characteristics of the emergency nurses and the mean scores of compliances with standard precautions

Variables	Nurses Compliance Mean Scores (n=138)											
	
	PCIP2P		UPD		DS		DSUA		DW		Total score	
	
	Mean	P-value	Mean	P-value	Mean	P-value	Mean	P-value	Mean	P-value	Mean	P-value
**Hospital name**	26.0	0.167	22.7	0.165	10.4		11.6	0.094				
King Khalid Hospital	26.2		22.8		10.6		11.9		3.4		74.2	
King Salman	26.4		22.6		10.9	0.332	11.5		3.7	0.232	75.2	0.080
Specialist Hospital	24.8		21.4		9.9		11.1		3.4		74.7	
Sharaf Urgent Care Hospital Maternity and Child Hospital									3.2		70.4	
**Age (years)**	26.2	0.013	22.7	0.313	10.6		11.7	0.354				
20 to < 35 years	26.7		22.9		10.5		11.6		3.5	0.381	75.8	
35 to < 45 years	25.1		22.1		10.3	0.701	11.4		3.5		75.2	0.085
More than 45 years									3.3		72.1	
**Gender**	25.5	0.079	22.9	0.262	10.8	0.239	11.6	0.930				
Male	25.7		22.4		10.4		11.6		3.4	0.527	75.2	0.211
Female									3.5		75.6	
**Nationality**	26.0	0.968	22.4	0.174	10.4		11.5	0.414				
Saudi	25.9		23.1		10.7	0.476	11.7		3.5	0.245	75.9	0.548
Non-Saudi									3.3		75.7	
**Educational level**	26.1	0.136	22.0	0.310	9.9		11.6	0.053				
Diploma in nursing	26.0		22.7		10.6		11.7		3.1		72.8	
Bachelor in nursing	24.0		21.7		9.7	0.141	10.3		3.5	0.284	74.5	0.102
Master degree in nursing									3.3		69.0	
**Profession carrier**	26.0	0.493	22.7	0.139	10.4		11.7	0.016				
Staff nurse	25.6		21.7		10.8	0.335	11.0		3.4	0.685	74.4	0.383
Profession career									3.5		72.7	

**Years of experience**	25.7	0.506	22.7	0.678	10.6		11.7	0.809				
Less than 5 years	25.9		22.4		10.5		11.6		3.5		74.1	
From 5 to 10 years	26.4		23.0		10.4	0.917	11.5		3.5	0.753	73.9	0.940
More than 10 years									3.3		74.8	

Data are expressed as percentage for categorical variables. The used tests were independent t-test and one-way analysis of variance ANOVA. PCIP2P: Prevention of Cross Infection from Person to Person; UPD: Use of Protective Device; DS: Disposal of Sharps; DSUA: Decontamination of Spills and Used Article; DW: Disposal of Waste

## Discussion

The current study aimed to assess the nurses' compliance with standard precautions during the COVID-19 pandemic at emergency departments, Hail, Kingdom of Saudi Arabia. The findings highlighted nurses' compliance with standard precautions. Regarding the socio-demographic data of the studied nurses; the results of the current study revealed that more than half of the studied nurses were in adult age group from 35 to 45 years old, most of them were females. Regarding the level of education and profession carrier, it was noticed that the majority of the nurses had bachelor in nursing and were staff nurses. Near half of them had working experience from 5 to 10 years. As regards to setting, more than half of the studied nurses were from King Khalid Hospital as it is the largest setting.

The results of this study showed that the overall total compliance rate of the studied nurses was optimal (more than 90%). The mean scores of standard precautions ranged from 3.1 to 3.9 out of 4, this finding is similar to the findings of previous studies who reported that the mean compliance with standard precautions among emergency nurses was 4.29 and 4.31 out of 5, respectively that used the same instrument (20, 21). In another study conducted among Saudi nursing students using a similar CSPS tool (17), results show the overall compliance rate of the students with standard precautions was moderate (60.1%). In addition, Saudi students have attained relatively higher scores on standard precautions compliance compared to Australian nursing students (59.8%) (22), but lower than students from Ghana (61.3%) (23), Italy (74.2%) (24) and Jordan (79.9%) (25). This finding may be because each country has a different curricular content on infection control, different teaching approaches, views, and perceptions of students.

The first dimension was assessed through seven items. The result indicates that the majority of nurses recorded optimal compliance scores (92.8%) with standard precaution in terms of prevention of cross infection from person to person during the period of COVID-19 pandemic. The result goes in the same line with the performance in South Korea regarding to the compliance with standard precaution (26). The compliance rate is slightly lower as regards to “using only water for hand washing” however it is satisfactory (85.0%). The CDC advises the public and health professionals to regularly wash their hands with ordinary soap and water for at least 20 seconds as it is considered the first-line preventive strategy of COVID-19 contamination. Alcohol-based hand sanitizers are recommended when soap and water are not accessible (27, 28). The second dimension is related use of protective devices that consisted of six items. The result of the current study showed that the overall rate of compliance represents is optimal and high satisfactory (94.2%), nevertheless, the result showed less compliant with reusing a surgical mask or disposable personal protective equipment (87.5%). This result was in agreement with other previous studies (29, 30).

Three items measure disposal of sharps as a dimension for measuring the compliance with standard precautions. The compliance rate is slightly lower (87.5%) however it is satisfactory. The result showed less compliant with “the sharps box is only disposed when it is full” however it is a satisfactory score, represents more than 77 percent, nurses are still requiring intensive training to increase knowledge about standards precaution related to the usage of needles after giving an injection, and the disposal of sharps box. This finding is supported by a study on this problem that reported inadequate needle safety precautions, low compliance with standard guidelines, and improper disposal of sharps among the health care workforce in a trauma care setting. This is despite the presence of an active infection control committee and the presence of posters stressing the need to comply with standard precautions (31).

Decontamination of spills and used article, disposal of waste are very important dimensions in measuring the compliance with standard precaution, the results of this study reflect high satisfactory compliance rates (96.5%). Additionally, the majority (87.5%) of emergency nurses did affirm that they place waste contaminated with blood, body fluids, secretion and excretion in red plastic bags irrespective of the patient's infection status. On the other hand, the results of the current study exist a statistically significant association between age and the dimension of prevention of cross infection from person to person, the highest mean was for the age group from 35 to 45 years (middle age). This result similar to previous finding which indicated that young age had a lower score of compliance with standard precautions (29). Furthermore, there is a significant association between professional category and decontamination of spills and used article, the highest mean of compliance noticed in nursing staff, this could be related to their direct contact with patients' equipment. The main strength of our study was its being the first study, which shows the prevalence of nurses' compliance with standard precautions during COVID-19 pandemic at emergency departments, Hail city, Saudi Arabia. Further longitudinal studies for continuous follow up and evaluation are recommended.

In conclusion, the compliance with standard precautions by emergency nurses was optimal (more than 90%). The mean compliance scores with the standard precautions could be associated with age and professional category. These results may contribute to direct strategies to encourage adherence to standard precautions of the MOH toward prevention of COVID-19 spread. Furthermore, continuous training program to enhance compliance with standard precautions among emergency nurses with continuous follow up and evaluation are recommended.
